# Energy consumption forecasting for laser manufacturing of large artifacts based on fusionable transfer learning

**DOI:** 10.1186/s42492-024-00178-3

**Published:** 2024-12-02

**Authors:** Linxuan Wang, Jinghua Xu, Shuyou Zhang, Jianrong Tan, Shaomei Fei, Xuezhi Shi, Jihong Pang, Sheng Luo

**Affiliations:** 1 State Key Lab of Fluid Power and Mechatronic Systems, Zhejiang University, Hangzhou, Zhejiang 310058 China; 2 Engineering Research Center for Design Engineering and Digital Twin of Zhejiang Province, Zhejiang University, Hangzhou, Zhejiang 310058 China; 3 State Key Lab of Materials Processing and Die & Mould Technology, Huazhong University of Science and Technology, Wuhan, Hubei 430074 China; 4 School of Marine Engineering Equipment, Zhejiang Ocean University, Zhoushan, Zhejiang 316022 China; 5 College of Business, Shaoxing University, Shaoxing, Zhejiang 312000 China; 6 College of Computer Science and Artificial Intelligence, Wenzhou University, Wenzhou, Zhejiang 325000 China

**Keywords:** Energy consumption forecasting, Large metal artifacts, Carbon peaking and carbon neutrality, Laser powder bed fusion, Fusionable transfer learning

## Abstract

This study presents an energy consumption (EC) forecasting method for laser melting manufacturing of metal artifacts based on fusionable transfer learning (FTL). To predict the EC of manufacturing products, particularly from scale-down to scale-up, a general paradigm was first developed by categorizing the overall process into three main sub-steps. The operating electrical power was further formulated as a combinatorial function, based on which an operator learning network was adopted to fit the nonlinear relations between the fabricating arguments and EC. Parallel-arranged networks were constructed to investigate the impacts of fabrication variables and devices on power. Considering the interconnections among these factors, the outputs of the neural networks were blended and fused to jointly predict the electrical power. Most innovatively, large artifacts can be decomposed into time-dependent laser-scanning trajectories, which can be further transformed into fusionable information via neural networks, inspired by large language model. Accordingly, transfer learning can deal with either scale-down or scale-up forecasting, namely, FTL with scalability within artifact structures. The effectiveness of the proposed FTL was verified through physical fabrication experiments via laser powder bed fusion. The relative error of the average and overall EC predictions based on FTL was maintained below 0.83%. The melting fusion quality was examined using metallographic diagrams. The proposed FTL framework can forecast the EC of scaled structures, which is particularly helpful in price estimation and quotation of large metal products towards carbon peaking and carbon neutrality.

## Introduction

Energy consumption (EC) usually refers to energy resources, among which electricity accounts for a large proportion, consumed by industrial equipment includes computer numerical control machine tool, laser manufacturing equipment, air separation unit, etc. Enhancing EC efficiency might contribute to carbon peaking and carbon neutrality, thereby addressing the global climate change which reflects mankind’s unremitting pursuit and exploration in sustainable development consensus. Additive manufacturing (AM), commonly referred to as three-dimensional (3D) printing, has garnered considerable attention owing to its capacity to produce intricate components and flexibility in producing personalized products [1]. AM technologies construct an object from the ground up, which endows AM with the capability to fabricate bespoke [2], topologically optimized [3], and intricately complex metallic constructs encompassing interior features [4]. However, replicating these attributes using conventional subtractive manufacturing methodologies is considerably complex [5]. Consequently, AM has been adopted in numerous fields, including buildings, biomechanical engineering, automotive, and aerospace, contributing to the rapidly increasing industry size [6]. The vigorous development of AM offers a novel manufacturing approach and leads to tremendous EC demand, raising concerns regarding sustainable production [7].

The prevailing global issues precipitated by climate change, diminishing biodiversity, and political instability have resulted in severe unpredictability concerning resource availability [8]. These conditions necessitate a comprehensive reassessment of conventional practices from the perspective of sustainability and resource management. To enhance AM sustainability, it is imperative to incorporate strategic sustainable design approaches across multiple facets, including the concept of AM equipment, process mapping, raw material development, and supply chain selection [9]. EC analysis is essential for the sustainable design of AM processes, which is the cornerstone for the optimization of fabrication variables to balance processing conditions and energy efficiency performance [10].

To achieve sustainable designs with feasible manufacturing parameters, the relationship between EC and producing variables has been investigated [11]. Energy efficiency is related to the input manufacturing parameters, including laser power, feed rate, and mass rate, for the laser-directed energy deposition process. Laser generating models can be improved to reduce the EC [12]. Hasan et al. [13] analyzed the interactive influences of the scanning speed, laser power, and feed rate on EC. The effects of these factors were qualitatively determined using regression analysis. The effects of infill patterns, which significantly affect the balance between part performance, EC, and material consumption, have also been studied [14]. The manufacturing duration was closely related to the total length of the hatching path, which considerably affected the EC. The type of fabricating material also significantly affects EC owing to the diverse requirements of the melting temperature and printing speed [15]. In summary, EC is associated with numerous factors, including AM categories, fabrication parameters, material types, and equipment. These intricate and interconnected factors make it difficult to construct a general and comprehensive framework for analyzing the EC of AM.

Laser powder bed fusion (LPBF)-based methodologies, e.g., selective laser melting (SLM), direct metal laser sintering (DMLS), and cold metal fusion, facilitate the fabrication of intricate components, particularly made with high-entropy alloys. During these processes, the desired cross-sections of the intended parts are meticulously scanned using a high-power laser source to selectively deposit thin layers of powdered material [16]. The ability to produce metal products makes LPBF a promising technology for industrial applications. However, it consumes significant energy for preparatory activities and melting materials. Papadakis et al. [17] evaluated and compared the EC of various preheating ways. The EC was calculated based on transient thermal simulation through the finite element method. Kota et al. [18] constructed an EC estimation model by analyzing the EC characteristics of the SLM process. The influences of the manufacturing parameters on energy usage were captured through an experimental investigation. However, these works summarized the overall EC but neglected the temporal variation in energy usage during the manufacturing process.

The development of artificial intelligence technologies, especially large language model (LLM), graph neural network, as superior analytical techniques provides a novel approach for energy forecasting [19, 20]. The powerful nonlinear fitting ability of neural networks enables temporal EC forecasting for complex systems and processes [21]. Schwung et al. [22] optimized the EC of hybrid production systems via actor-critic reinforcement learning. Actuator behavior-dependent functions were adopted to predict EC. Wang et al. [23] exploited simple structures to approximate the EC of patterns fabricated in complex structures. The EC was predicted through deep learning by matching similar infill patterns. The generalization ability of deep learning-based techniques is significantly related to the training data. Thus, forecasting EC under various conditions with fewer computing resources remains a significant topic.

Digital twin (DT) maps the structure, state, characteristics, and functionalities of a physical entity into a virtual entity in a digital form. Through the utilization of programmable logic control and evolutionary information of the virtual entity, DT enables the prediction of the physical entity, facilitating interactive feedback between the virtual and physical entities [24]. Torvi et al. [25] visualized the complex energy footprint via modified Sankey diagrams to provide a clear and intuitive representation for the analysis of energy flow in AM processes. Denkena et al. [26] integrated a DT with real process data and simulation outcomes to optimize manufacturing processes and reduce EC. The real-time simulation and analysis of AM processes enable the optimization of EC steps and assist in energy-efficient design [27].

Based on previous studies [28–32], specifically the reconfigurable transfer learning technique [31], an EC forecasting framework for the laser melting manufacturing process of metal artifacts based on fusionable transfer learning (FTL) is proposed. To achieve a prospective analysis of the temporal EC, a general EC analysis framework was constructed by dividing the LPBF process into three main steps. Innovatively, the manifolds of metal artifacts are decomposed into scanning trajectories, which can be further transformed into fusionable information to overcome the limitations of geometric structures in EC forecasting. An operator network consisting of parallel trunk and branch components is constructed. The fusionable information from both components was fused to obtain the final output, whose deviation from the measured electrical power was minimized. The discretized fabrication inputs and parallel network structure endow the proposed FTL with the generalizability of scalable structures, specifically from scale-down to scale-up structures.

## Methods

### General paradigm for EC in laser manufacturing process

The EC refinement is the basis of energy efficiency optimization. The LPBF manufacturing process can be categorized into three main stages: preprocessing, fabrication, and postprocessing. EC during the preprocessing and postprocessing stages is closely related to external factors and varies significantly owing to different operators. Thus, EC during the fabrication process is investigated. The total EC, $\:{E}_{t}$, can be considered as the accumulation of subprocesses in the manufacturing process

1 $$\:\begin{array}{c}E_t=\sum\limits_{n=1}^NE_n=\sum\limits_{n=1}^Np_nt_n\end{array}$$

where $\:{E}_{n}$ represents the EC of the $\:{n}^{th}$ subprocess; $\:{p}_{n}$ and $\:{t}_{n}$ are the power and runtime of the $\:{n}^{th}$ subprocess, respectively.

Specifically, the fabricating subsystems can be divided into many parts, including controlling, driving, melting, and auxiliary systems. The calculated EC varies according to the decomposition of the manufacturing system. To make the EC framework more general and compatible, fabrication was divided into three steps: preheating, melting, and post-heating. EC can be formulated as a combinatorial sum of the following steps

2 $$\:\begin{array}{c}E_t=E_{pre}+E_{mel}+E_{pos}\end{array}$$

where $\:{E}_{pre}$, $\:{E}_{mel}$, and $\:{E}_{pos}$ are the ECs of the preheating, melting, and post-heating processes, respectively.

The EC of each step can be further expanded as the product of power and runtime

3 $$\:\begin{array}{c}E_t=P_{pre}t_{pre}+P_{mel}t_{mel}+P_{pos}t_{pos}\end{array}$$

where $\:{P}_{pre}$, $\:{P}_{mel}$, and $\:{P}_{pos}$ are the operating power of the preheating, melting, and post-heating steps, respectively, and $\:{t}_{pre}$, $\:{t}_{mel}$, and $\:{t}_{pos}$ are the corresponding runtimes.

The printing progress rate is denoted by the normalized height, $\:{h}_{n}$. $\:{h}_{n}$ of the *i*^th^ layer can be defined as

4 $$\:{h}_{n}=\frac{{z}_{i}}{{z}_{b}}\:{h}_{n}\in\:\left(\text{0,1}\right]$$

where $\:{z}_{i}$ represents the cumulative layer height and $\:{z}_{b}$ denotes the total height of the manifold.

### EC forecasting via FTL

Ideally, the operating power of each step of fabrication can be obtained by adding the power of each working component. Nevertheless, the operating power is affected by different material properties, manufacturing parameters, product structures, and voltage fluctuations. These factors render it difficult to accurately confirm the operating power at each step of the fabrication process.

Generally, the operating power of each step, $\:{P}_{s}$, can be considered as function of the fabrication arguments, including material properties $\:{\varvec{F}}_{M}$, manufacturing parameters $\:{\varvec{F}}_{P}$, and geometry structures $\:{\varvec{F}}_{G}$. The formulation of function $\:{f}_{P}$ is determined by equipment parameters $\:{\varvec{\theta\:}}_{e}$.

5 $${P}_{s} = {f}_{P}\left(\varvec\theta_{e}\right)\left(\varvec{F}_{M}, \varvec{F}_{P}, \varvec{F}_{G}\right)$$

**Fig. 1:**
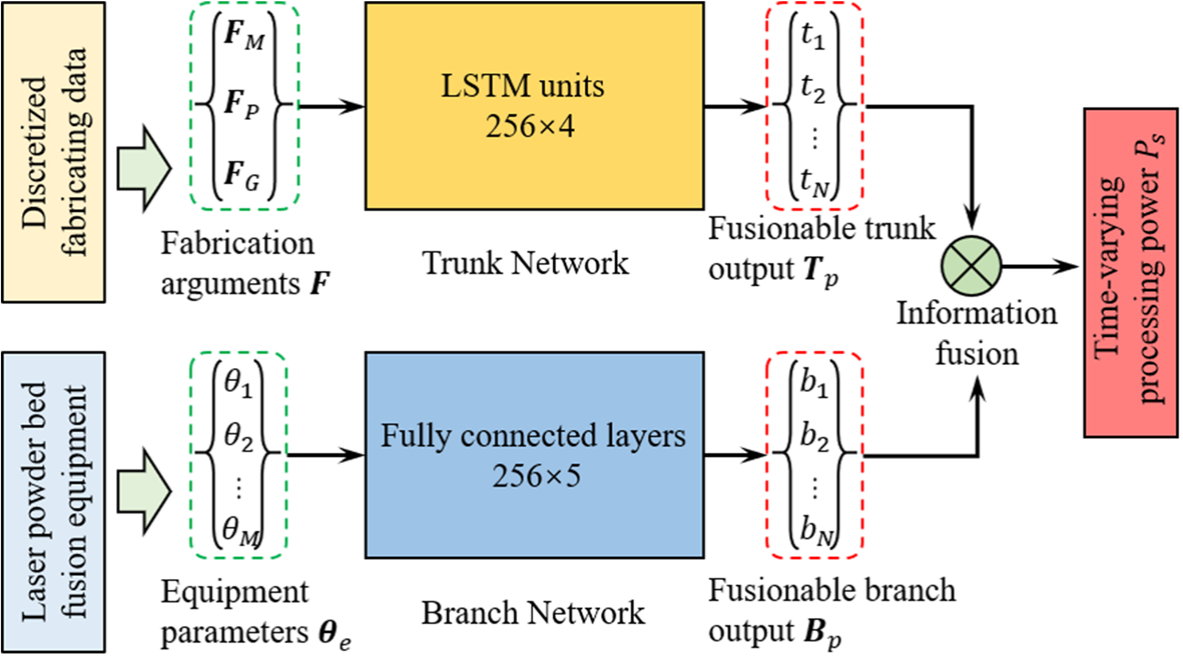
Logical structure and information fusion operation of parallel neural networks for EC forecasting

EC forecasting can be formulated as the prediction of the power of each step according to the fabrication arguments and equipment parameters. Operator learning networks are adopted and trained for various steps to forecast the power and runtime (Fig. 1).

The operator neural network comprises two parts: trunk and branch networks. The trunk network considers the fabricating variables as inputs and the branch network handles the equipment parameter inputs. The fusionable outputs of the trunk and branch were combined with a dot product operation. Fully connected layers were implemented as the branch network. The long short-term memory structure was adopted as the trunk network for predicting power owing to its sequential character.

The runtime of the melting step can be accurately calculated based on the scanning trajectories and speed. Thus, the runtime of melting step, $\:{t}_{mel}$, can be confirmed according to Eq. 6.

6 $$\:\begin{array}{c}t_{mel}=\sum\limits_{i=1}^I\frac{l_i}{v_i}\end{array}$$

where $\:{l}_{i}$ and $\:{v}_{i}$ denote the scanning trajectories length and speed, respectively. Generally, the power of the melting step varies when scanning and melting different parts of a product, including border filling, contour filling, area hatching, and support hatching.

### Power measurement principles and EC forecasting

The measurement of the real-time operating power consists of two parts: the measurement of voltage $\:u\left(t\right)$ and current $\:i\left(t\right)$. For a device supplied by an alternating current (AC), effective voltage $\:U$ and current $\:I$ within one cycle $\:T$ can be confirmed according to Eqs. 7 and 8.

7 $$\:\begin{array}{c}U=\sqrt[2]{\frac{1}{T}{\int}_{0}^{T}{u}^{2}\left(t\right)dt}\end{array}$$

8 $$\:\begin{array}{c}I=\sqrt[2]{\frac{1}{T}{\int}_{o}^{T}{i}^{2}\left(t\right)dt}\end{array}$$

Active power $\:P$ within one cycle $\:T$ can be calculated using the integral of the instantaneous power.

9 $$\:\begin{array}{c}P=\frac{1}{T}{\int}_{0}^{T}u\left(i\right)i\left(t\right)dt\end{array}$$

Accordingly, the constructed neural network can be optimized by minimizing the mean squared error (MSE) loss, as formulated in Eq. 10.

10 $$\:\begin{array}{c}MSE=\sum\limits_{i=1}^{{N}_{T}}\frac{{\left({P}_{S}-P\right)}^{2}}{{N}_{T}}\end{array}$$

where $\:{N}_{T}$ is the number of sampled power values.

The overall EC, $\:E$, can be further computed from the production of power and sampling interval $\:t$

11 $$\:\begin{array}{c}E=\sum\limits_{i=1}^{{N}_{T}}\frac{\left({P}_{i}+{P}_{i-1}\right)\times\:t}{2}\end{array}$$

**Fig. 2:**
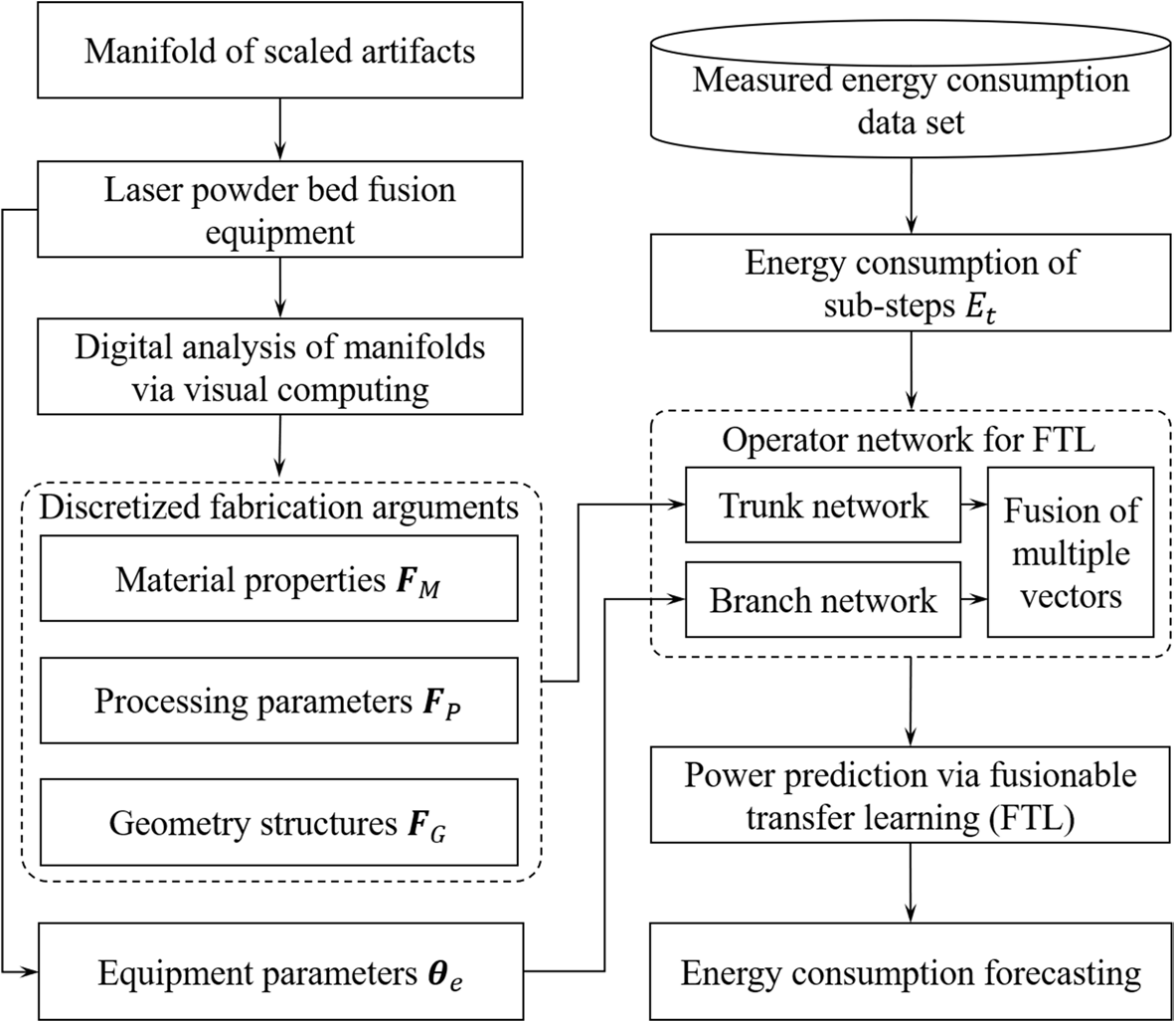
Overall logical flowchart of the proposed FTL-based energy forecasting

Figure 2 displays the overall logical flowchart of the proposed FTL for energy forecasting with scalability under various producing conditions. The operator network was trained using the measured EC information. To predict the EC of the scaled artifacts that may be fabricated with different devices, discretized fabrication arguments were obtained through digital analysis. The parallel-arranged networks were fine-tuned to investigate the impact of fabrication variables and equipment on EC through transfer learning. The intermediate information of both components (trunk and branch networks) was fused as the final output, which realized EC forecasting via FTL.

## Results and Discussion

### Digital analysis of artistic dragon totem via information fusion visualization

#### Layered volume of the manifolds to be fabricated

**Fig. 3:**
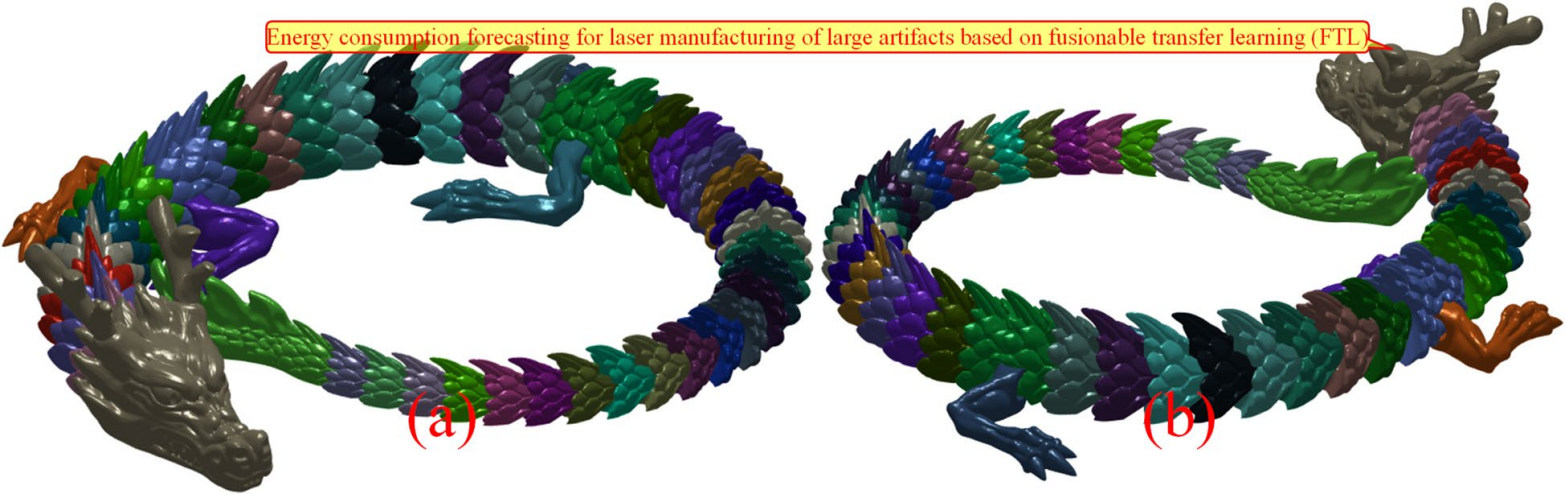
Overall diagram of a colorful artistic dragon totem from different views. **a** is the front view and **b** is the back view

An artistic dragon totem, usually symbolizes good luck in Oriental culture, consisting of 44 entities, was adopted as an example because of its sophisticated structure (Fig. 3). By default, the length unit is millimeters (mm). The total surface area is $\:{S}_{object}$= 71474.1863 mm^2^, total volume of the enclosed manifold is $\:{V}_{object}$= 80672.7190 mm^3^, and the specific surface area is 0.8860 mm^-1^. The net mass when stainless steel was used is 645.3818 g. An increase in decimal precision is induced by the meshing of rational manifolds, which is significant during the digital calculation process.

Voxel is one of the most widely used forms of discrete expression which offers a solution for simplifying complex 3D structures. The voxelization is essential for conducting FTL, which is beneficial for reducing computational pressure when decomposing an artifact into discretized fabrication arguments.

**Fig. 4:**
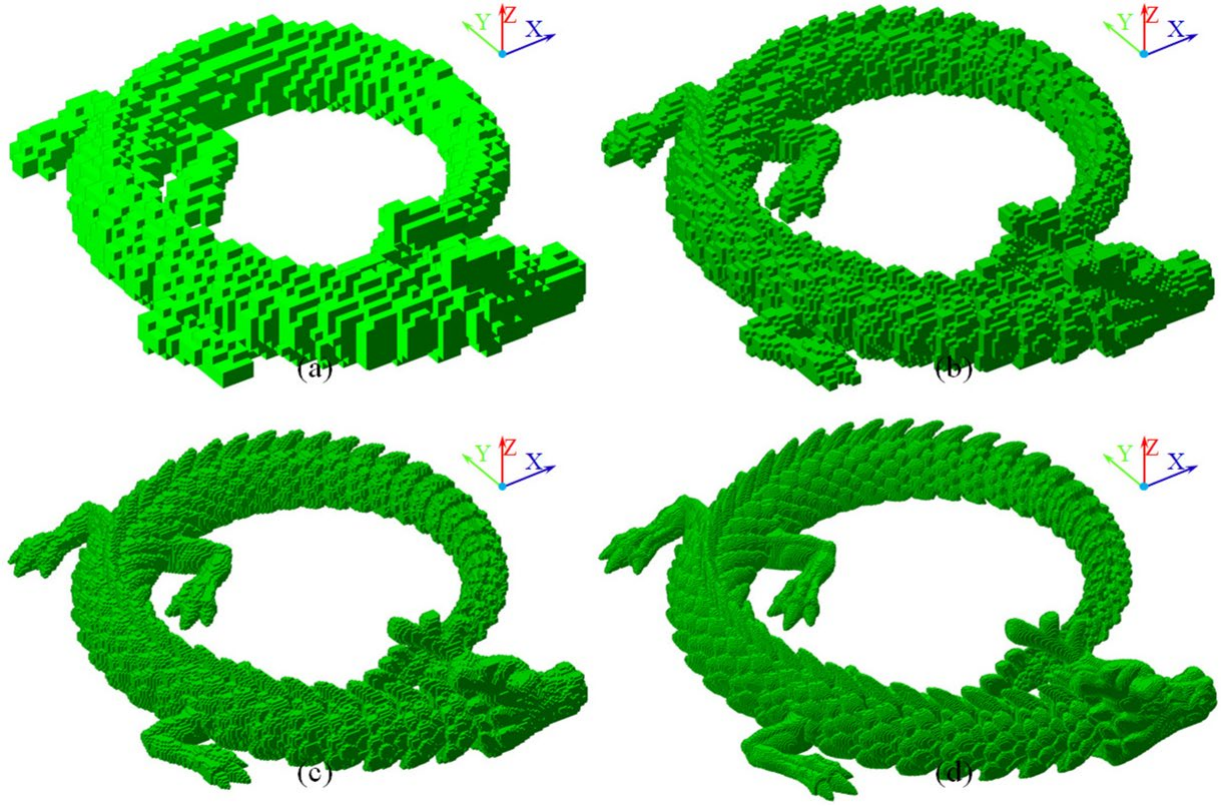
Tunnel-free voxelization of artifact for FTL with various resolutions. **a**, **b**, **c**, and **d** are the voxelizations obtained under voxelizing resolutions of 64^3^, 128^3^, 256^3^, and 512^3^, respectively

A tunnel-free voxelization of the artifacts at various resolutions is constructed in Fig. 4. The number of voxels is 3781 for the voxelization in Fig. 4a, and the geometrical size of a single voxel is $\:2.7549\times\:2.8239\times\:3.0195\:$ mm^3^. The number of voxels is 30,888 for the voxelization in Fig. 4b and the size of a single voxel is $\:1.3906\times\:1.3895\times\:1.4539\:$ mm^3^. The number of voxels is 254,206 for the voxelization in Fig. 4c and the size of a single voxel is $\:0.6855\times\:0.6893\times\:0.6887$ mm^3^. The number of voxels is 2,005,539 for the voxelization in Fig. 4d and the size of a single voxel is $\:0.3436\times\:0.3433\times\:0.3474\:$mm^3^.

**Fig. 5:**
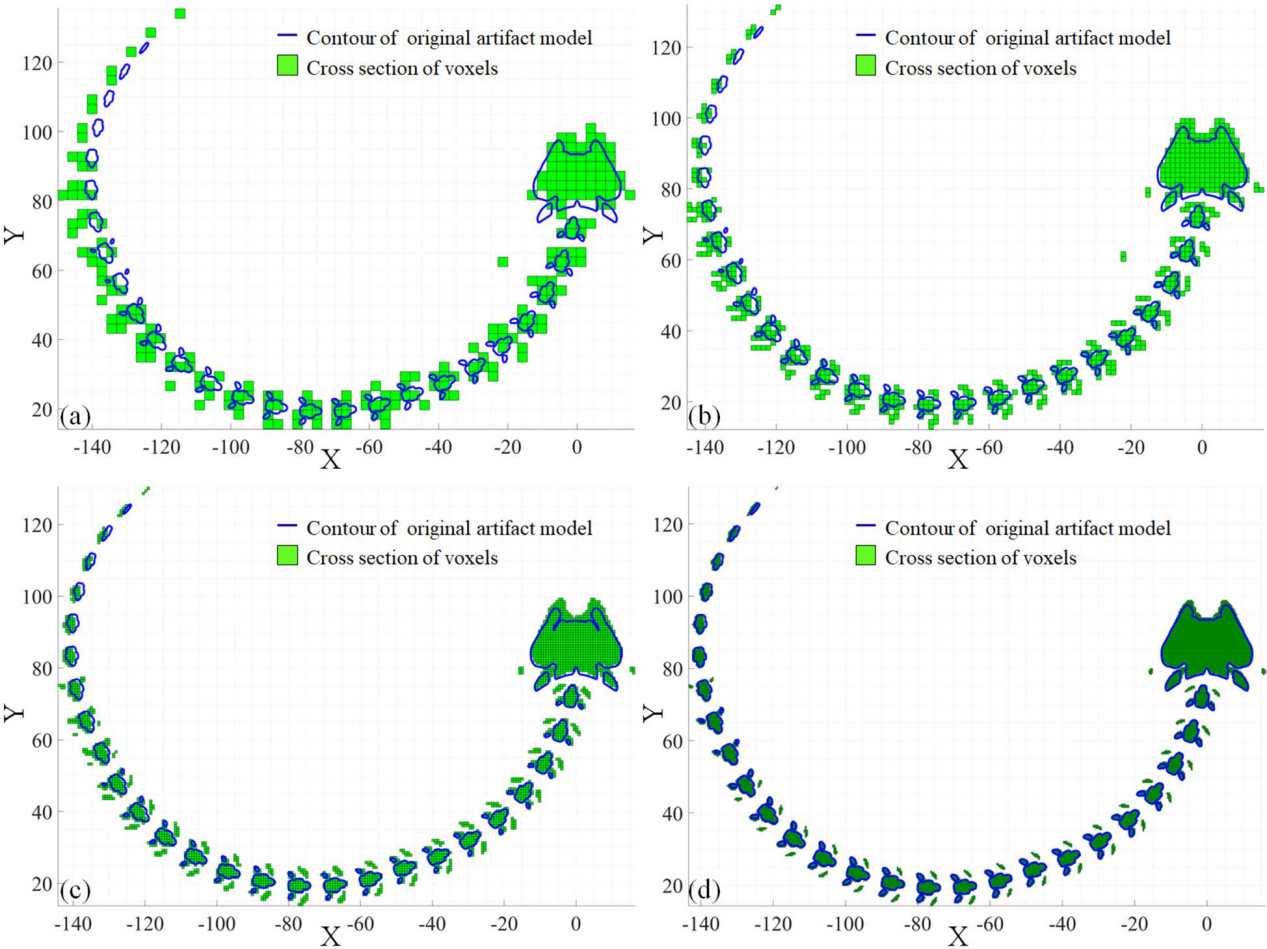
Cross sections of the voxelization and contours of the artifact. **a**, **b**, **c**, and **d** illustrate the outcomes under 64^3^, 128^3^, 256^3^, and 512^3^, respectively

A cross-sectional comparison of the voxelization and the original artifact is illustrated in Fig. 5. The contour was obtained by extracting the intersection lines of the artifact with a plane parallel to XY-plane whose normalized height *h*_*n*_, is 50%. The range of *h*_*n*_, area, area error, and portion of area error of the voxels in Fig. 5a are 49.2063%–50.7937%, 1863.3541 mm^2^, 1009.5602 mm^2^, and 54.1797%, respectively. The range of *h*_*n*_, area, area error, and portion of the area error of the voxels in Fig. 5b are 49.6063%–50.3937%, 1481.8952 mm^2^, 628.1013 mm^2^, and 42.3850%, respectively. The range of *h*_*n*_, area, area error, portion of the error area of the voxels in Fig. 5c are 49.8039%–50.1960%, 1181.6068 mm^2^, 398.8698 mm^2^, and 33.7566%, respectively. The range of *h*_*n*_, area, area error, portion of the error area of the voxels in Fig. 5d are 49.9022%–50.0978%, 1066.0966 mm^2^, 212.3027 mm^2^, and 19.9140%, respectively.

The voxelization resolution significantly affects the approximation accuracy of the original 3D structure, leading to impacts on the decomposition of the original artifact. Accordingly, inaccurate discretized fabrication arguments, which are closely related to the decomposition accuracy, damage the prediction accuracy and efficiency of the proposed FTL.

**Fig. 6:**
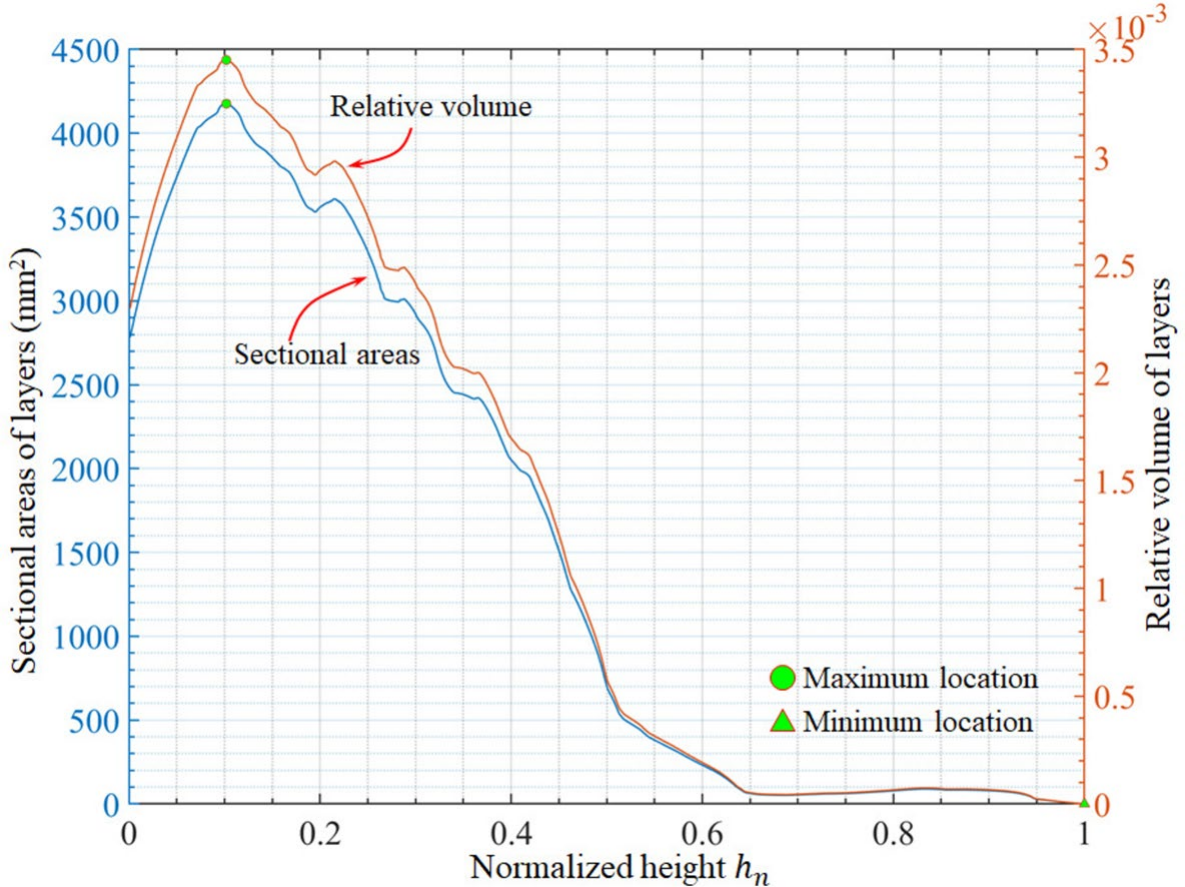
Variation of areas and volumes of sliced layers during laser manufacturing

Figure 6 presents the variations in the cross-sectional areas and relative volumes of the sliced layers. The total area, maximum layer area when $\:{h}_{n}=10.19\%$, minimum layer area, average, and standard deviation are $\:1.21\times\:{10}^{6}$ mm^2^, $\:4.18\times\:{10}^{3}$ mm^2^, 0.29 mm^2^, 1541.57 mm^2^, and 1555.01 mm^2^, respectively. The total volume, maximum layer volume when $\:{h}_{n}=10.19\%$, and relative proportion are $\:1.21\times\:{10}^{5}$ mm^3^, 417.62 mm^3^, and 0.35%, respectively. The minimum layer volume is 0.03 mm^3^, whose relative proportion is $\:2.42\times\:{10}^{-5}$%, close to zero. The average volume of the layers is 154.16 mm^3^ and the standard deviation is 155.50 mm^3^.

#### Preprocessing of artistic dragon totem with scaled structures

**Fig. 7:**
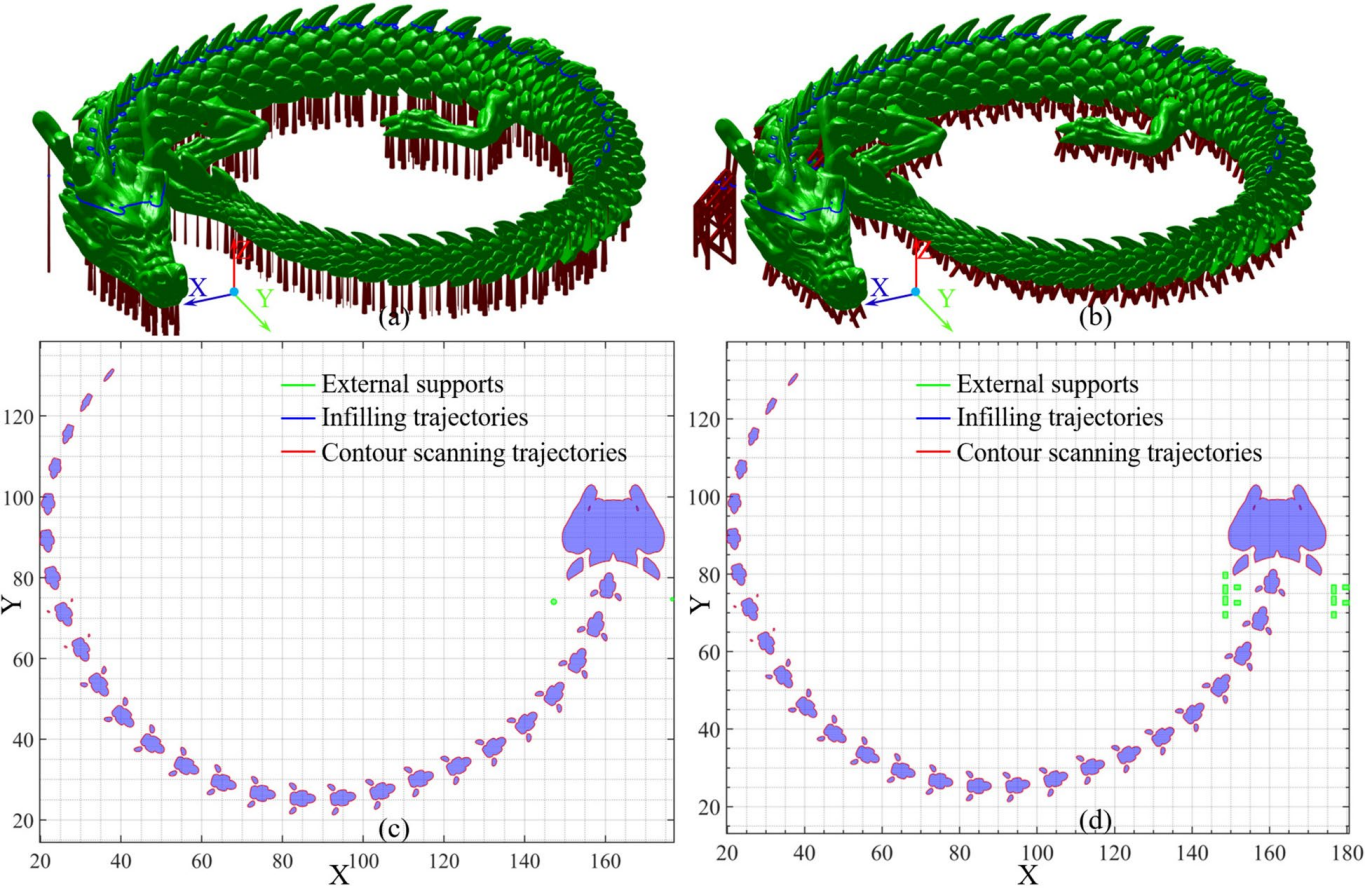
Artistic dragon totem with various external support structures

External supports are essential auxiliary structures that guarantee manufacturability, resulting in inevitable EC. An artistic dragon totem with various external support structures is displayed in Fig. 7. The dragon totem with column supports is displayed in Fig. 7a, in which the surface area, total volume, support portion and specific surface area of the external supports are 17699.9 mm^2^, 4568 mm^3^, 0.0057%, and 3.8748 mm^-1^, respectively. A dragon totem with rectangular supports is shown in Fig. 7b, in which the surface area, total volume, and support mass portion and specific surface area of the external supports are 17891.1 mm^2^, 4083 mm^3^, and 0.0051%, and 4.3819 mm^-1^, respectively.

**Fig. 8:**
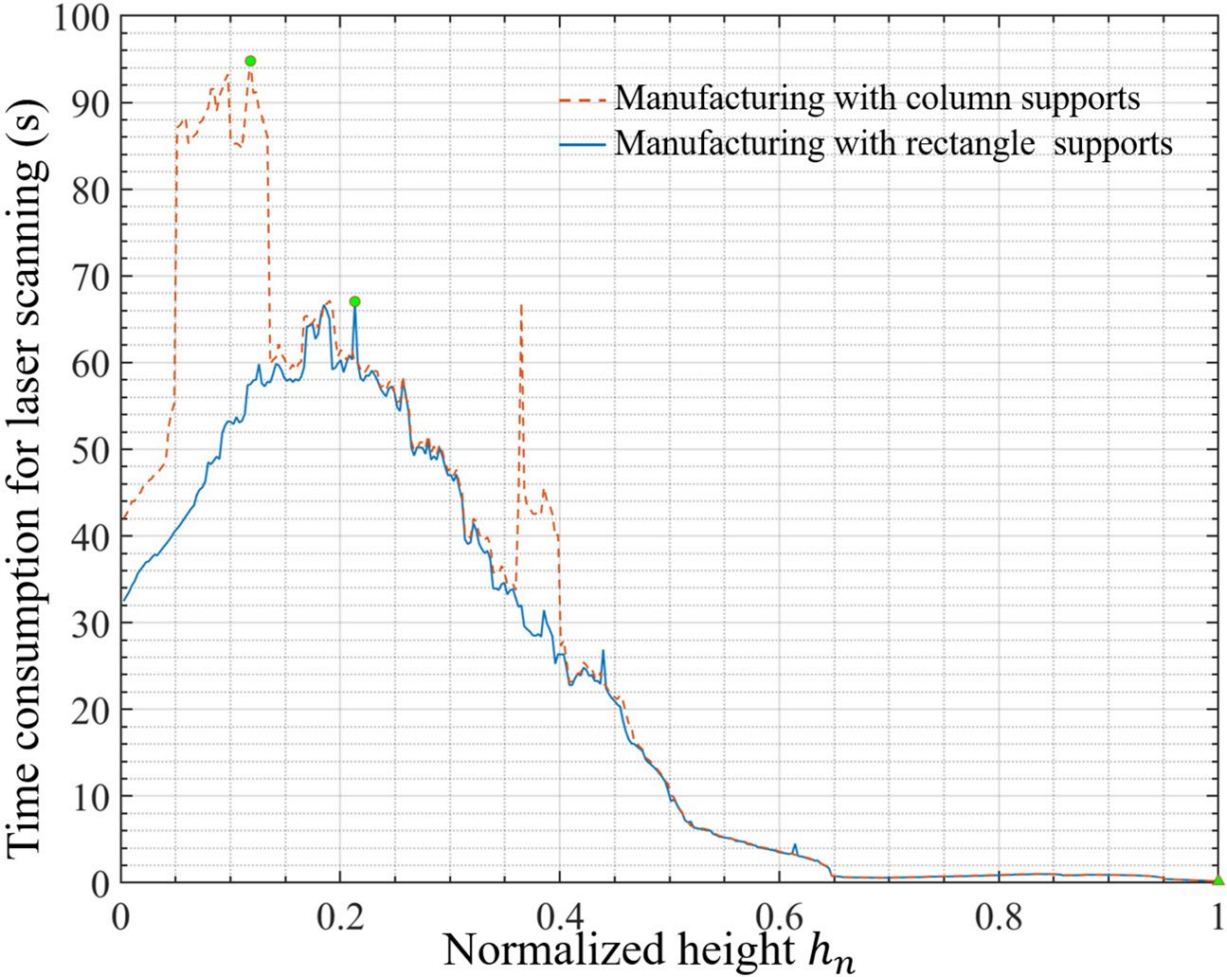
Time consumption for fabricating slice layers of artistic dragon totem with various external support structures

The runtime for forming the layer can be further confirmed for conducting the FTL, as well as the time required to form an artistic dragon totem with different support structures is demonstrated in Fig. 8. With external column support as illustrated in Fig. 7a, the maximum time consumption for fabricating slices occurred when $\:{h}_{n}$= 21.34%, minimum occurred when $\:{h}_{n}$= 1, standard deviation, variance, median, and mean are 67.04 s, 0.18 s, 22.82 s, 520.75 s^2^, 9.6059 s, and 21.91 s, respectively. The total time consumption for melting is 8521.40 s. With external rectangle support as illustrated in Fig. 7b, the maximum time consumption for fabricating slices occurred when $\:{h}_{n}$ = 11.8252%, minimum occurred when $\:{h}_{n}$ = 1, standard deviation, variance, median, and mean are 94.79 s, 0.1807 s, 29.30 s, 858.42 s^2^, 10.10 s, and 26.53 s, respectively. The total melting time is 10,319 s. It should be noted that the manufacturing time depends on the working capacity of the mother machine.

#### Virtual manufacturing of artistic dragon totem via information fusion visualization

**Fig. 9:**
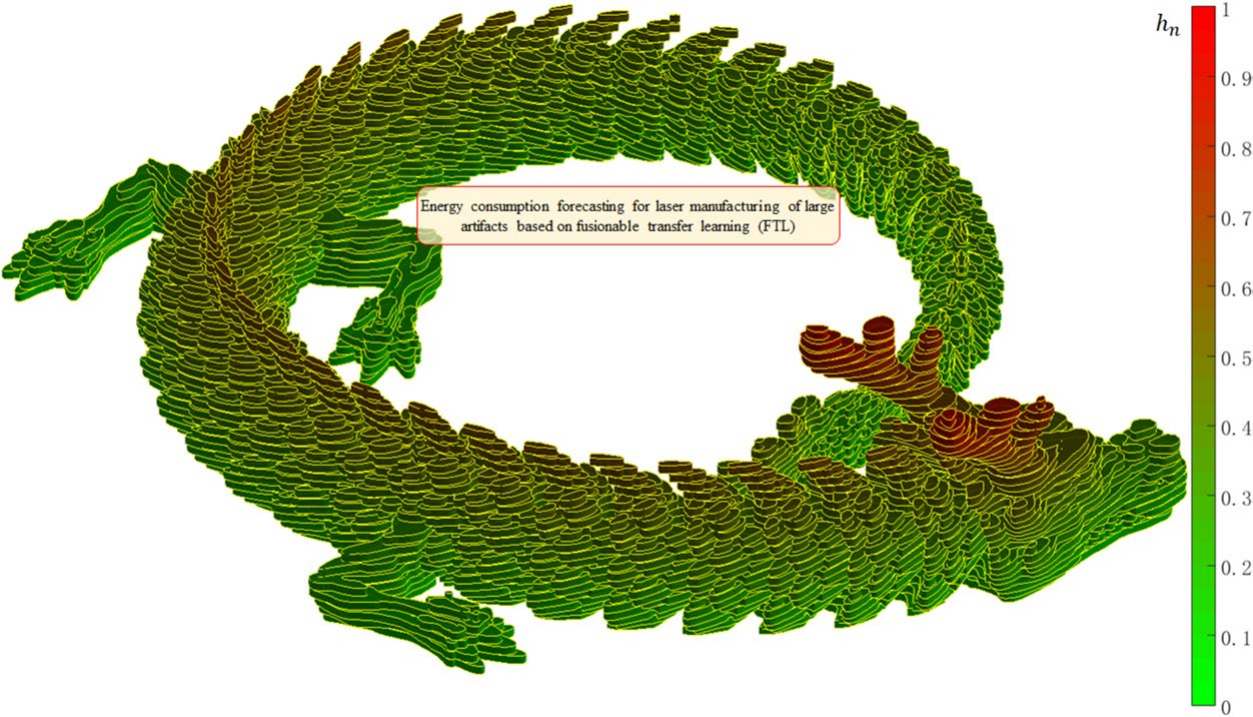
The slicing visualization of an artistic dragon totem via hierarchical DT technique

The effectiveness of the obtained manufacturing parameters was verified using a visualized hierarchical DT technique. As displayed in Fig. 9, it demonstrates the slicing visualization of an artistic dragon totem with merely 30 layers. A DT-based virtual prototype was constructed based on the cross-sectional contours of the layered artifacts.

**Fig. 10:**
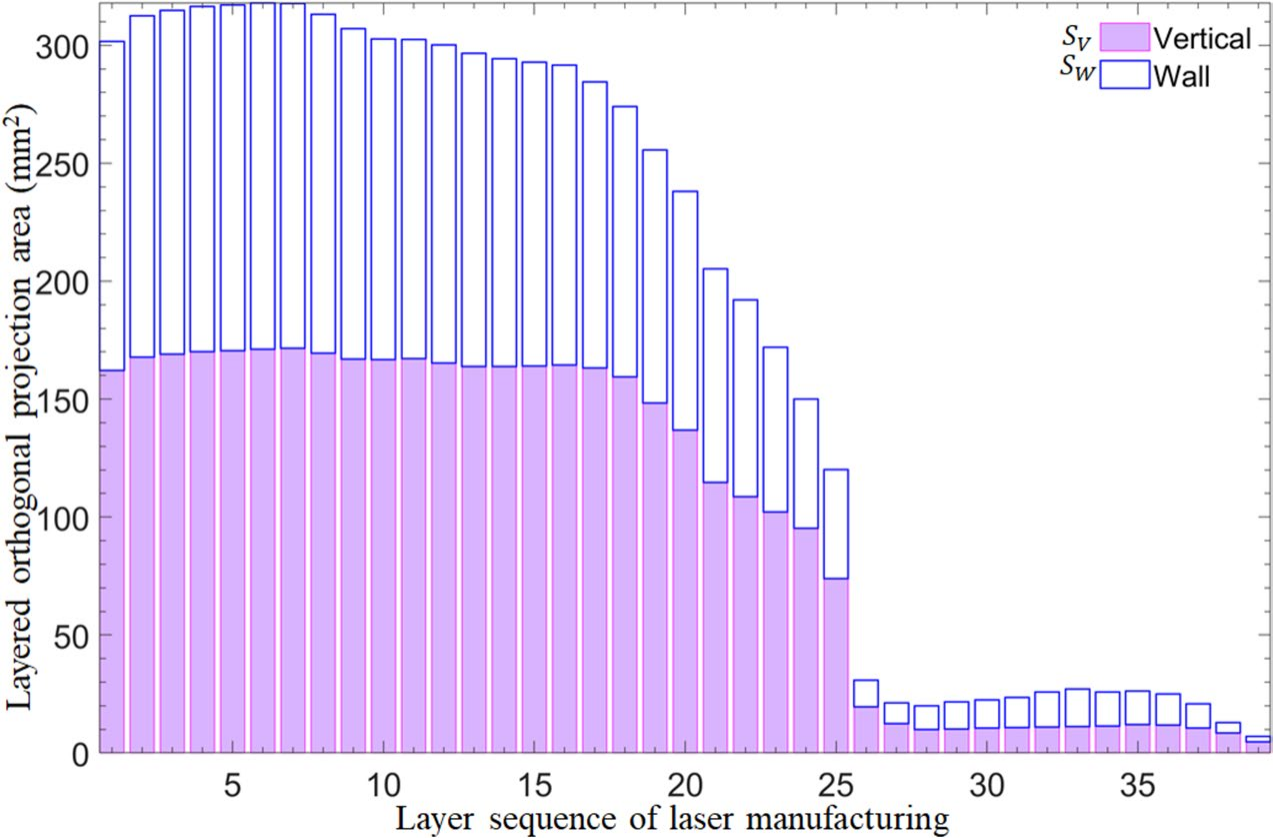
Orthogonal projected area of 3D manifold when the number of layers is 40

The gradient of the projected area must be controlled to reduce relative volume error. Figure 10 exhibits the orthogonal projected area of 3D manifold. *S*_*V*_ is the projected area on the vertical plane, whereas *S*_*W*_ is the projected area on the plane of width. The details are presented in Table 1.

**Table 1:** Orthogonal projected area of 3D manifold

Orthogonal projected area		40 layers
Orthogonal projected area *S*_*V*_ in vertical plane	Maximum (mm^2^)	1.715129e + 02
	Minimum (mm^2^)	4.710675
	Sum (mm^2^)	3931.2561
	Mean (mm^2^)	100.8014
	Standard deviation (mm^2^)	71.6942
	Variance (mm^4^)	5140.0564
Orthogonal projected area *S*_*W*_ in wall plane	Maximum (mm^2^)	1.467872e + 02
	Minimum (mm^2^)	2.278211
	Sum (mm^2^)	3168.9121
	Mean (mm^2^)	81.2542
	Standard deviation (mm^2^)	58.2945
	Variance (mm^4^)	3398.2467

### Physical experiments for verifying the effectiveness of FTL in EC forecasting

#### Physical fabrication of large metal artifact via LPBF

Manufacturing large metals usually takes more energy than making small non-metals. An industrial LPBF device was adopted to conduct physical experiments to verify the effectiveness of the proposed FTL. The accuracy of the FTL was quantitatively evaluated by comparing the predictions and measurements. The calibration curve and relative error were adopted as evaluation indices.

**Fig. 11:**
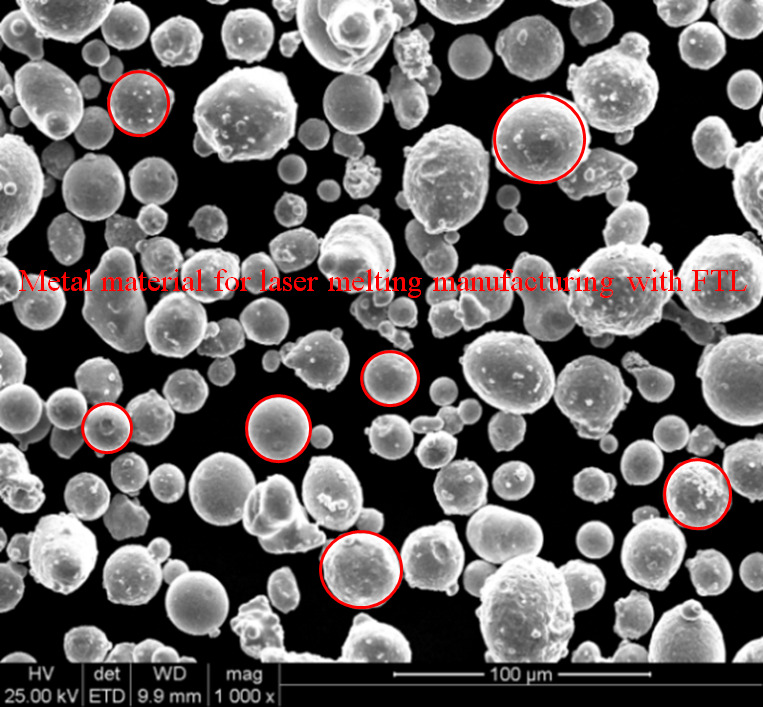
Scanning electron microscope image visualization of 316 L stainless steel powder

LPBF devices can rapidly manufacture products and omit complex processes, which is unavoidable with conventional techniques (such as casting). The manufacturing materials can be stainless steel, titanium alloy, high-temperature alloy, nickel-cobalt alloy, and die steel. A physical fabrication experiment was conducted using powdered 316 stainless steel. The powder size range is ø15–ø52 µm, the grain size when the cumulative distribution proportion in the system reaches 10%, 50%, and 90% are D10 = 23.1 μm, D50 = 35.3 μm, and D90 = 53.3 μm, respectively. It contains material elements with various mass fraction (Wt%): Chromium Cr (16.79), Mo (2.42), Ni (10.66), Si (1.00), Mn (0.20), S (0.011), P (0.025), and O (0.025). The mechanical properties of the materials are listed in Table 2. The additive Mo improved the corrosion resistance. The formed parts are mainly composed of austenite, which has good toughness and low strength. A scanning electron microscope image of the stainless steel powder is shown in Fig. 11.

**Table 2:** Mechanical properties of 316 L stainless steels

Steel material	Density (×1000 kg/m^3^)	Poisson’s ratio	Elastic modulus (Gpa)	Tensile strength (Mpa)	Yield strength (Mpa)
316 L	8	0.27–0.30	192–210	480	170

**Fig. 12:**
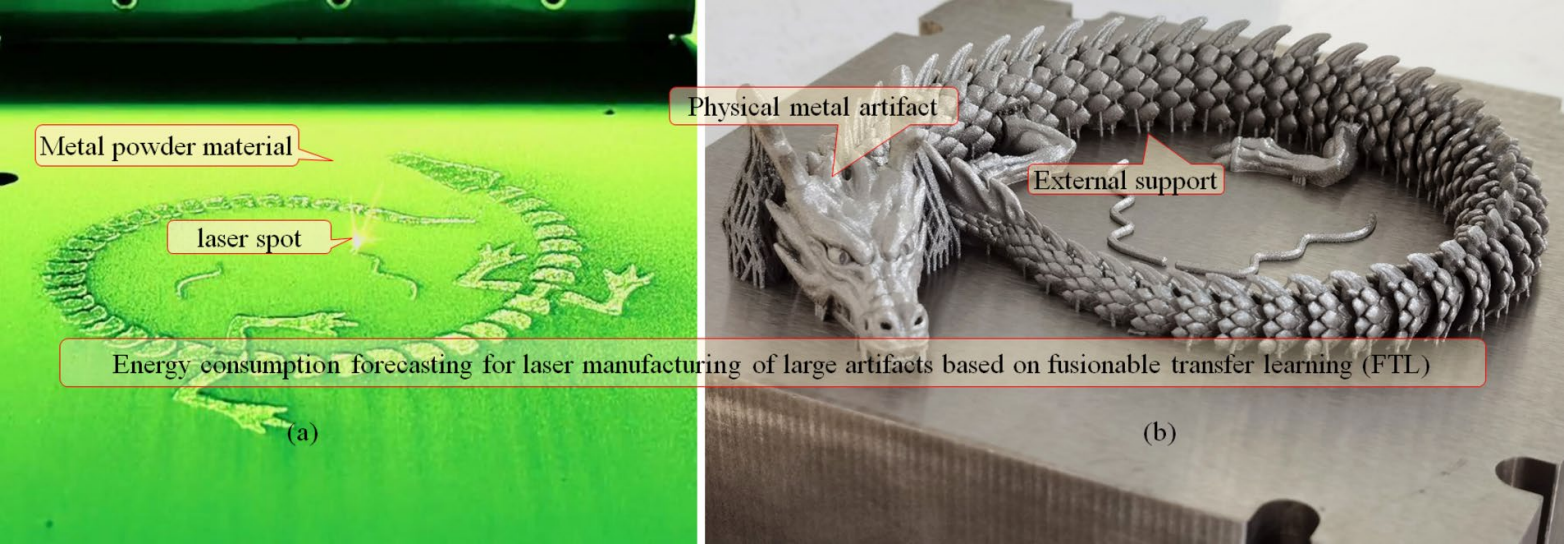
Physically fabricated metal artifact via LPBF. **a** The intermediate manufacturing process; **b** The overall fabricated large metal artifact

A physically fabricated artistic dragon totem is displayed in Fig. 12. The mechanical properties of the fabricated products can surpass those of castings and even reach the level of forging. The size of the adopted device was $\phi\,170\times\:200$ mm. The layer thickness can be adjusted within 0.02–0.1 mm. A fiber laser with 500 W is equipped and the scanning speed of the galvo scanning system can exceed 15 m/s, with the repeatability of $\:\pm\:\:0.02$ mm. The laser diameter is within 70–100 $\:{\upmu}\text{m}$. The dimensional accuracy is 0.05–0.1 mm for products less than 100 mm, and $\:\pm\:\:1\text{\%}$ for those over 100 mm. The power supply requirement is an AC of 220 V/50 Hz. Nitrogen was used as a protective gas to isolate the oxygen and prevent oxidation. The scan speed, average thickness, laser absorptivity, and substrate temperature were 500 mm/s, 0.03 mm, 0.7, and 20 ℃, respectively. The ambient temperature at a relative humidity of 55% is 20 ℃.

#### Energy forecasting for fabricating metal artifact with various supports via FTL

**Fig. 13:**
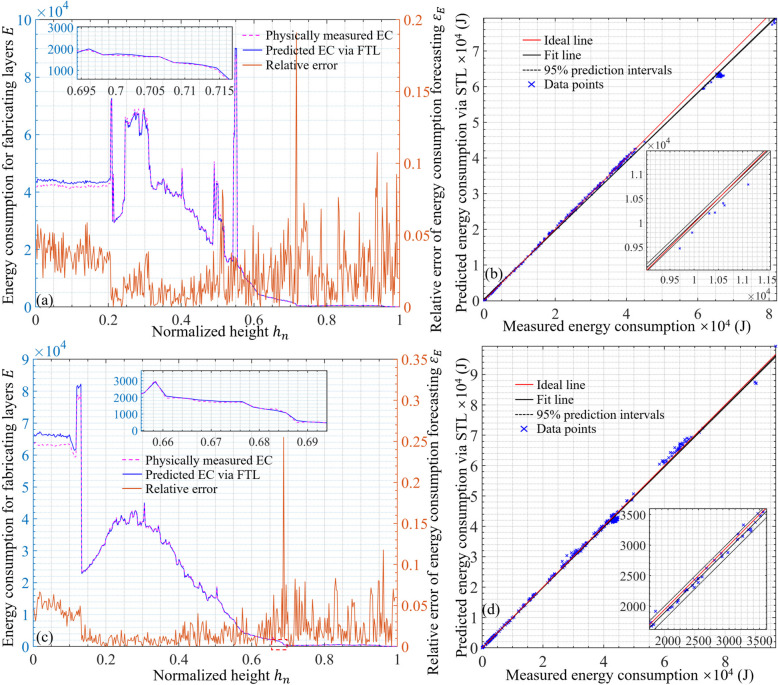
Perceptual prediction of large component manufacturing process via proposed FTL. **a** and **b** represent the energy consuming forecasting with column supports; **c** and **d** are with rectangle supports

The FTL-based EC prediction program can be completed in C language. The perceptual prediction outcomes of EC during the AM process via the FTL are demonstrated in Fig. 13. Figure 13a plots the predicted and measured ECs with the column supports. For each layer, the maximum and minimum measured values are $\:9.9332\times\:{10}^{4}$ J and 64.11 J at 36.9072% and 99.79%, respectively. The maximum and minimum predicted values are $\:9.6641\times\:{10}^{4}$ J and 69.17 J at 36.9072% and 98.76%, respectively. In total, the measured and predicted EC of fabrication process are 1.1769 × 10^7^ J (equivalent to 3.2692 kW·h) and 1.1863 × 10^7^ J (equivalent to 3.2953 kW·h), respectively. Figure 13b illustrates the calibration curve of the perceptual prediction. Linear equation *y* = 0.9936*x* − 37.0782 was derived to visually characterize the perceptual prediction accuracy distribution. For all layers, the MSE of the regression of the calibration curve was $\:7.2956\times\:{10}^{5}$ J, the root mean square error (RMSE) was 854.1414 J, the ordinary R^2^ was 0.9985, and the maximum absolute error among all cases was 3343.3970 J.

Figure 13c plots the predicted and actual measured EC with the rectangular supports. For each layer, the maximum and minimum measured values are $\:8.7554\times\:{10}^{4}$ J and 64.1052 J at 54.75% and 99.7938%, respectively. The maximum and minimum predicted values are $\:9.0199\times\:{10}^{4}$ J and 69.17 J at 55.17% and 98.76%, respectively. In total, the measured and predicted EC of fabrication process are 1.1670 × 10^7^ J (equivalent to 3.2417 kW·h) and 1.1766 × 10^7^ J (equivalent to 3.2683 kW·h), respectively. Figure 13d illustrates the calibration curve of the perceptual prediction. Linear equation *y* = 0.9926*x* − 20.0199 was derived to visually characterize the perceptual prediction accuracy distribution. For all layers, the MSE of the regression of the calibration curve was $\:4.8212\times\:{10}^{5}$ J, the RMSE was 694.3493 J, the ordinary R^2^ was 0.9990, and the maximum absolute error among all cases was 2255.4639 J. The quantitative evaluation of the energy forecasting is listed in Table 3.

Generally, the relative error was maintained below 8%. The average and overall predictions had higher accuracies than the predictions of the peak values. The relative prediction error was comparatively lower for the layers that consumed more energy, indicating the significant application potential of the FTL in manufacturing processes with high EC.

**Table 3:** Relative error of energy forecasting via FTL with various structures

Value		Measured (J)	Prediction (J)	Relative error (%)
Fabricating with column supports (Fig. 13a)	Maximum	9.9332 × 10^4^	9.6641 × 10^4^	2.71
	Minimum	64.11	69.17	7.90
	Average	2.4266 × 10^4^	2.4459 × 10^4^	0.80
	Overall	1.1769 × 10^7^	1.1863 × 10^7^	0.80
Fabricating with rectangle supports (Fig. 13b)	Maximum	8.7554 × 10^4^	9.0199 × 10^4^	3.02
	Minimum	64.10	69.17	7.90
	Average	2.4111 × 10^4^	2.4310 × 10^4^	0.83
	Overall	1.1670 × 10^7^	1.1766 × 10^7^	0.82

#### Metallographic diagram of fabricated metal artifact

**Fig. 14:**
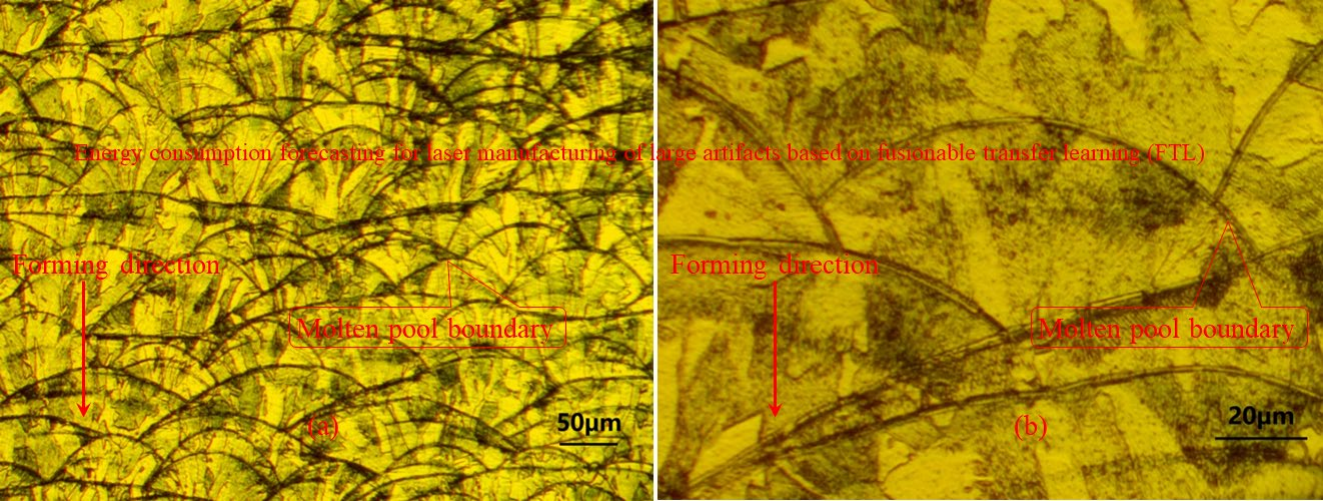
Metallographic diagram visualization of fabricated metal microstructures

A metallographic diagram of the fabricated metal product at different magnification rates was obtained to observe the microstructure of the layer melting fusion, as illustrated in Fig. 14. Metallurgical microscopy is widely applied for the detection of semiconductors, flat panel display, circuit packages, electric subgrade plates, metal/ceramic components, and precision dies. Notably, the gravity direction in Fig. 14 is upward, owing to the orientation of the metallographic sample. The minimum graduation in fine movement of focusing system is 2 μm and the size of objective table is 192 mm × 141 mm, which can be adjusted within the range 50 mm × 40 mm. The illumination system offers a 12 V 50 W halogen lamp, as well as a field stop and aperture stop. Marangoni convection may occur inside the molten pool because of the gradient in the surface tension of the molten pool.

## Conclusions

### EC forecasting method for laser melting manufacturing of metal artifacts based on FTL is proposed

To address the EC forecasting of AM, a FTL-based method was proposed. To accurately predict the EC in the fabrication stage of LPBF, regardless of the structures of the products, the general combinatorial function of the EC was categorized into substeps. An operator network inspired by LLM with a parallel structure was then constructed by considering the discretized fabrication arguments as inputs. The proposed FTL is helpful for improving the energy efficiency and price estimation, specifically for large metal products towards carbon peaking and carbon neutrality.

### FTL was conducted to forecast EC of metal artifacts with various external support structures

A general EC paradigm was first constructed by categorizing the overall process into three main parts. The digital manifold of metal artifacts was analyzed from various aspects, including layer slice areas, projected areas, and scanning trajectories. An operator neural network consisting of trunk and branch parts was constructed and trained, so as to fit the relationships between the electrical power responses and fabricating variables. The fusionable information obtained from the network components was fused as the final output. The FTL could boost conceptual design iteration of large artifacts.

### The effectiveness of proposed FTL in forecasting EC of scalable artifact structures was verified through physical experiments

The effectiveness of the proposed FTL in forecasting the EC of the laser-scanning AM process was verified through physical experiments. The artistic dragon totem was physically fabricated via LPBF, and the ECs were obtained simultaneously. The FTL was implemented to predict the EC during the fabrication process. The prediction errors of the scalable structures were evaluated quantitatively using calibration curves. The melting-fusion quality of the fabricated material was confirmed using metallographic diagrams.

In future research, the EC of diverse manufacturing technologies will be investigated in advanced mechanical fields, including spatial bending pipes, in-orbit 3D printing, or more. The proposed FTL could be applied to forecast the EC under variable manufacturing conditions.

## Data Availability

The data will be available upon non-profit response.

## Abbreviations

FTL: Fusionable transfer learning
LPBF: Laser powder bed fusion
AM: Additive manufacturing
EC: Energy consumption
SLM: Selective laser melting
DMLS: Direct metal laser sintering
DT: Digital twin
3D: Three-dimensional
MSE: Mean squared error
AC: Alternating current
RMSE: Root mean square error
LLM: Large language model
